# Diagnostic accuracy of screening tools for depression and anxiety in cervical dystonia

**DOI:** 10.1016/j.parkreldis.2025.107891

**Published:** 2025-05-26

**Authors:** Davide Martino, Mehrafarin Ramezani, Steven Bellows, Brian D. Berman, Florence Ching-Fen Chang, Jeanne Feuerstein, Victor Fung, Gamze Kilic Berkmen, Irene A. Malaty, Claire MacIver, Scott A. Norris, Kathryn J. Peall, Joel S. Perlmutter, Sarah Pirio Richardson, Laura J. Wright, Zahra Goodarzi, Hyder A. Jinnah

**Affiliations:** a Department of Clinical Neuroscience, Cumming School of Medicine & Hotchkiss Brain Institute, University of Calgary, Calgary, Alberta, Canada; b Parkinson’s Disease Center and Movement Disorders Clinic, Baylor College of Medicine, Houston, TX, USA; c VCU Parkinson’s and Movement Disorders Center, Virginia Commonwealth University School of Medicine, Richmond, VA, USA; d Department of Neurology, Westmead Hospital, Sydney, New South Wales, Australia; e Department of Neurology, University of Colorado School of Medicine, Aurora, CO, USA; f Department of Neurology, Rocky Mountain Regional VA Medical Center, Aurora, CO, USA; g Sydney Medical School, Westmead Clinical School, Faculty of Medicine and Health, University of Sydney, Sydney, New South Wales, Australia; h Department of Neurology, Emory University School of Medicine, Atlanta, GA, USA; i Department of Neurology, University of Florida, Fixel Institute for Neurological Diseases, Gainesville, FL, USA; j Cardiff University Brain Research Imaging Centre (CUBRIC), Neuroscience and Mental Health Innovation Institute, Cardiff University, Cardiff, CF24 4HQ, United Kingdom; k Departments of Neurology and Radiology, Washington University School of Medicine, St. Louis, Missouri, USA; l Division of Psychological Medicine and Clinical Neurosciences, Neuroscience and Mental Health Innovation Institute, Cardiff University, Cardiff, CF24 4HQ, United Kingdom; m Departments of Neurology, Radiology, Neuroscience, Physical Therapy and Occupational Therapy, Washington University School of Medicine in St. Louis, St. Louis, Missouri, USA; n Department of Neurology, University of New Mexico Health Sciences Center and New Mexico VA Health Care System, Albuquerque, NM, USA; o Department of Neurology, Washington University School of Medicine, St. Louis, Missouri, USA; p Division of Geriatric Medicine, Department of Medicine, Community Health Sciences and Clinical Neurosciences, Cumming School of Medicine, Hotchkiss Brain Institute and O’Brien Institute of Public Health, University of Calgary, Calgary, Alberta, Canada; q Department of Neurology, Human Genetics, and Pediatrics, Emory University School of Medicine, Atlanta, GA, USA

**Keywords:** Cervical dystonia, Depression, Anxiety, Screening, Accuracy, Rating scales

## Abstract

**Introduction:**

Despite their high prevalence and impact, depression and anxiety are not routinely screened for, and accuracy of screening procedures is unknown in adult-onset dystonia. We evaluated accuracy parameters of selected self-rated scales for depression and anxiety in patients with idiopathic cervical dystonia (CD).

**Methods:**

Two-hundred-and-ten patients with idiopathic CD were recruited from 10 movement disorders centers from the US, Canada, Australia, and UK. At the end of each botulinum toxin cycle, participants were administered the Adult Standard Mini-International Neuropsychiatric Interview (MINI) as reference standard for depression and anxiety. Participants completed 8 self-administered index instruments (2 for depression, 2 for anxiety, and 4 combining screening for both). Sensitivity, specificity, positive and negative predictive values, covariate-adjusted area under the receiver operating characteristic curve (AUC), and likelihood ratios were calculated for all instruments.

**Results:**

On the MINI, 8.6 % (100 % female) fulfilled criteria for current major depressive disorder and 10.5 % (91 % female) fulfilled criteria for any current disorder amongst panic, social anxiety or generalized anxiety disorders. For depression screening, all tools had an AUC higher than 0.80, with the two most accurate being the BDI-II (AUC 0.91, sensitivity 87.5 %) and the HADS-Depression (AUC 0.88, sensitivity 93.7 %). For anxiety screening, the only instrument showing clinical usefulness was the HADS-Anxiety (AUC 0.82, sensitivity 86.3 %).

**Conclusion:**

Current major depression can be screened in CD with high degree of accuracy using different self-administered scales, whereas existing screening tools for anxiety perform worse. Dystonia-specific instruments are less accurate than scales developed for the general population.

## 1. Introduction

Depression and anxiety can impair quality of life, increase disability and predict early retirement for patients with cervical dystonia (CD) more than motor symptoms [1,2]. In CD, mood symptoms do not respond consistently to botulinum toxin therapy, which highlights their different treatment profile and need for early and accurate detection [3, 4].

Despite increased awareness of non-motor symptoms in CD, the lack of an operational framework including screening and treatment of such symptoms contributes to incomplete care and patient dissatisfaction [5]. Limited attention from clinicians and researchers to non-motor symptoms in dystonia contributes to the dearth of clinical trials for neuropsychiatric symptoms. Yet, open label studies and a pilot trial suggest that cognitive behavioral therapy (CBT) can provide symptomatic benefit in more than 60 % of CD patients [6].

Establishing clinically applicable, affordable screening instruments, will help better define study cohorts and lead to more treatment options. Diagnostic structured interviews like the Mini International Neuropsychiatric Interview (MINI) are considered the reference standard for depression and anxiety screening but are lengthy and not feasible in most clinical settings. Presently, the screening accuracy of easily implemented self-rating instruments in dystonia is largely unknown and, as a result, not part of standard of care. The present study aims to evaluate the sensitivity and specificity of self-administered rating instruments for depression and anxiety that have most frequently been used in previous research relating to patients with CD.

## 2. Patients and methods

This is a multi-center and cross-sectional prospective study. Data collection was planned before administration of both the index and reference standard. Patients were recruited between January 2023 and January 2024 from 10 movement disorders outpatient clinics from 4 countries: University of Calgary, University of New Mexico, Virginia Commonwealth University, Baylor College of Medicine, University of Colorado, University of Florida, Washington University School of Medicine in St. Louis, Emory University, Westmead Hospital/University of Sydney and Cardiff University. Consecutive patients were screened for eligibility by their movement disorders neurologist at the participating clinical center. Inclusion criteria were a diagnosis of adult-onset, idiopathic, isolated CD according to the Movement Disorders Society Task Force Criteria, with no restrictions placed on sex, current/past non-motor symptoms profile and ongoing treatment. We excluded patients who started or had a dose change of an antidepressant or anxiolytic treatment in the preceding 8 weeks to minimize treatment confounds in the early phase of treatment. Data were collected within 1 week before or after a botulinum toxin treatment session, to minimize confounding effects from the cyclic efficacy of botulinum toxin on mood.

The study protocol was approved by the Calgary Health Research Ethical Board (CHREB) of the University of Calgary (REB19-2017). Data were collected locally and collated in a centralized REDCap database coordinated in Calgary. Participants were enrolled after signed informed consent.

*General data.* We recorded for each participant age, sex, years of education level, and ongoing active treatments for depression and anxiety. *Psychiatric assessments.* Participants were interviewed by the same training-certified examiner at each site using the Adult Standard Mini-International Neuropsychiatric Interview (MINI) [7] as a reference standard diagnostic instrument for depression and anxiety. Within 3 days and unaware of their MINI results, participants completed 8 self-administered rating instruments (index tests: 2 for depression, 2 for anxiety, and 4 combining screening for depression and anxiety), selected from self-rated tools previously used in neurological studies or specifically developed for people with dystonia (Table 1). Their order of completion was pseudo-randomized across participants at each site.

Based on our meta-analyses [8,9] and data from the Dystonia Coalition cohort [10], our study sample of 210 patients provided a power of 92 % to detect a sensitivity of 0.70 or greater for each of the tested screening instruments, at an alpha of 0.05 with 95 % confidence intervals. Missingness of data was handled applied the multiple imputation method. Sensitivity, specificity, positive and negative predictive values were calculated for all instruments at each score cut-off using, as reference standard, the presence or absence of a mood or anxiety disorder on the MINI diagnostic interview, administered by the same qualified interviewer at each site. Optimal score cut-offs were chosen based on the highest possible combination of sensitivity and specificity, calculating the Youden’s J index (which is defined as sensitivity + specificity − 1). Positive and negative likelihood ratios were calculated using binomial regression models, representing the clinical utility of the test by its relative impact on post-test probability. The area under the curve of receiver operator characteristic (ROC) curve analysis was used to assess optimal cut-off values for the different depression and anxiety scales, and their discriminative value in the diagnosis of depression and anxiety using the MINI as reference. For each covariate of interest (age, sex, disease duration, presence/absence of active therapies for mood or anxiety disorders, and recruiting center), we calculated covariate-adjusted ROC curves, which represent a weighted average of the covariate-specific ROC curves, with weights corresponding to the proportion of cases in each covariate group.^32^ Data analyses were carried out using Stata version 14.0 statistical software.

Study reporting has followed the STARD 2015 Reporting Guideline for accuracy studies (full checklist in Supplementary File).

## 3. Results

Two-hundred-and-twenty-one participants with adult-onset idiopathic isolated CD were recruited, but 11 had to be excluded from data analysis due to incomplete data collection; 210 completed the study protocol. We did not encounter screen failures. Mean age (± standard deviation) was 64 ± 11.4 years; 158 (75.2 %) were females. Sixty-two (29.5 %) were on a stable treatment plan for depression, anxiety or both, primarily using pharmacotherapy alone (49/210, 23.3 %). Supplementary Table 1 summarizes demographic features and mental health treatments for all participants, and recruitment breakdown by centre.

Using the MINI reference standard screening instrument, 8.6 % (100 % female) fulfilled criteria for current major depressive disorder (MDD; used for our accuracy analyses), 27.6 % (86.2 % female) fulfilled criteria for past history of MDD (which was recurrent in 55 %), and 10.5 % (91 % female) fulfilled criteria for any current disorder amongst panic, social anxiety or generalized anxiety disorders. Given the marked predominance of females in our study cohort with current depressive or anxiety disorder, we could not adjust our ROC curve analyses by sex.

Using the optimal cut-off points based on ROC curves, the prevalence of depression was 18.6 % (BDI-II, cut-off point 19), 28.6 % (PHQ-9, cut-off point 7), 23.8 % (HADS-D, cut-off point 7), 29.5 % (TWSTRS-PSYCH, cut-off point 7), 30.5 % (CDIP-58 selected questions, cut-off point 6), and 24.3 % (DNMS-Quest items 5–8, cut-off point 3). The area under the ROC curve was higher than 0.83 for all depression screening tools (Table 2). The two most accurate tools were: the BDI-II, which had a covariate-adjusted area under the ROC curve of 0.91 (95 % CI 0.85–0.99), and sensitivity and specificity reported at the optimal cut-off point of 87.5 % and 87.1 %, respectively (Supplementary Figure 1); and the HADS-Depression, which had a covariate-adjusted area under the ROC curve of 0.88 % (95 % CI 0.83–0.94), and sensitivity and specificity reported at the optimal cut-off point of 93.7 % and 82 %, respectively (Supplementary Figure 2). The performance of the other tools is summarized in Table 2.

Using the optimal cut-off points based on ROC curves, the prevalence of anxiety was 34.3 % (BAI, cut-off point 12), 66.6 % (STAI, cut-off point 89), 38.6 % (HADS-A, cut-off point 7), 34.8 % (TWSTRS-PSYCH, cut-off point 6), 36.2 % (CDIP-58 selected questions, cut-off point 5), and 41.4 % (DNMS-Quest items 5–8, cut-off point 2). Only the HADS-A yielded a covariate-adjusted area under the ROC curve higher than 0.8 (0.82 %, (95 % CI 0.73–0.91), and sensitivity and specificity reported at the optimal cut-off point of 7 % of 86.3 % and 67 %, respectively (Supplementary Figure 3). The performance of the other tools is summarized in Table 2.

Of the three tools containing items on both depression and anxiety (TWSTRS-PSYCH, CDIP-58 selected questions and DNMS-Quest items 5–8), only TWSTRS-PSYCH and CDIP-58 selected questions yielded a covariate-adjusted area under the ROC curve higher than 0.8 when screening for depression, anxiety or both, although their sensitivity and specificity values were all below 76 % (Table 2).

Adjustment for age, disease duration, presence/absence of active therapies for mood or anxiety disorders and recruiting center did not yield any significant effect on the performance of any of the rating instruments explored. Secondary analyses of the ROC curves adjusting independently for the presence of each of the four main groups of pharmacological therapies for mood or anxiety disorders listed in Supplementary Table 1 also did not yield significant effects on the performance of any of the instruments. A secondary analysis adjusted for focal vs. segmental/multifocal status of CD showed a significant effect of this covariate on the performance of DNMS-Quest in screening for depression (higher area under the curve for segmental/multifocal CD). A similar trend was observed for TWSTRS-PSYCH (Supplementary Figure 4). There were no adverse events recorded from performing the index tests or the reference standard.

## 4. Discussion

Our analysis identified several self-rating instruments that accurately screened depression in CD, thus offering several valid options for use across clinical practice and research. For broad screening, where optimization of sensitivity is preferred, the HADS-D presents as the favored instrument, with a sensitivity close to 94 %. The preferred cut-off for the HADS-D in patients with CD is consistent with the standard cut off for clinically meaningful depression in the general population [11]. Given the global distribution of the HADS, and its translation into at least 30 languages, clinical administration for CD patients should be facile to implement.

In clinical and research settings that favor specificity, the BDI-II appears to be favored. The BDI-II presents many advantages, including ease of use, low reading level, and excellent reliability [12]. The identified BDI-II optimal cut-off of 19 for our CD sample separates mild from moderate or severe depression also in the general population. Moreover, unlike the HADS-D, it screens specifically for suicidal ideation, which was present in 19/210 (9 %) of our cohort. Even though the BDI-II assesses physical symptoms which may not be specific for depression, the high specificity observed in our study mitigates this concern. The BDI-II has an estimated time of self-administration of 5–10 min, slightly longer than that of the HADS (2–5 min). Finally, both the HADS and the BDI-II are under copyright, which may limit their use in some settings.

We also identified the HADS-A as the best anxiety screening instrument for CD. Therefore, the HADS can perform high accuracy screening for both depression and anxiety in CD. The optimal cut-off (>7) for HADS-A is lenient, and separates, based on its traditional scoring interpretation, normal levels of anxiety from mild pathologic anxiety.^33^ This is reflected also by its specificity (67 %), which remained relatively low.

The screening performance of the instruments specifically developed for idiopathic dystonia (TWSTRS-PSYCH, DNMSQuest and CDIP-58) was sub-optimal, and more influenced-in the case of DNMSQuest and TWSTRS-PSYCH-by whether CD was focal or segmental/multifocal. Further refinement would be necessary before the TWSTRS-PSYCH and the other dystonia-specific instruments explored here can be recommended as high-accuracy screening instruments for clinically relevant depression and anxiety in CD.

Our clinic-based population of patients with CD is, for its demographic features, representative of the general population of patients affected by this condition and periodically followed up by a specialist clinic for botulinum toxin administration. Almost 30 % of our participants were already actively treated for depression or anxiety, reflecting that all the recruiting sites were specialist movement disorder clinics. Our findings could guide clinical screening for these psychiatric symptoms with a likely greater gain in accuracy within general Neurology services.

With respect to limitations, we did not include patients not followed up regularly for botulinum toxin injections, hence potentially excluding those with low disease severity, refractoriness to, or lack of financial coverage for, this treatment. Future studies should also investigate whether the accuracy of these scales changes when explored at the time of botulinum toxin peak effect, to measure the influence of fluctuations in motor symptoms and perceived disability on scale accuracy. We could not evaluate whether reported sex influenced the accuracy of the scales explored due to the large predominance of females among our patient population, and particularly in the subgroup with depression or anxiety according to the MINI. The MINI was chosen due to ease of use, shorter time of administration and good interrater reliability and convergent validity. However, its rigid structure may have contributed, together with the inclusion of patients undergoing stable treatment, to the relatively lower prevalence of current depressive and anxiety disorders compared to previous observational studies in CD, most of which applied rating scales rather than diagnostic interviews. Not having assessed the current severity of motor symptoms of CD, we cannot rule out completely an influence of motor severity on the accuracy of individual rating instruments. Finally, due to the multicentric design, an assessment bias based on the use of multiple evaluators administering the MINI cannot be totally ruled out. However, all evaluators underwent the same training on the diagnostic interview, and our analyses were adjusted for recruiting center.

In summary, current major depression can be screened in CD with valid and accurate self-rating tools such as HADS-D and BDI-II, whereas the screening performance for current clinically relevant anxiety is suboptimal for all the instruments most frequently applied in observational studies of CD. As a result of these findings, further investigation of the construct of anxiety in CD can guide whether other existing instruments may be more suitable screening tools, or if a new instrument needs to be developed.

## Supplementary Material

Supplementary Table 1

Fig. S1

suppl1

Fig. S2

Fig. S3

Fig. S4

## Figures and Tables

**Table 1:** Basic characteristics and properties of the clinimetric instruments administered in the study.

**REFERENCE STANDARD**	
**Adult Standard Mini-International Neuropsychiatric Interview (MINI)**	Brief structured diagnostic interview assessing the 17 most common psychiatric disorders in DSM-5 and ICD-10. Questions read verbatim and phrased to allow only “yes” or “no” answers; examples provided to facilitate responses. Administration duration: 25–30 min.
**DEPRESSION SCREENING INSTRUMENTS**	
**Patient Health Questionnaires 9 and 2**	PHQ-9: nine questions (scored 0–3), one for each of the DSM-IV criteria for major depression. The cut-off point of 10 for the PHQ-9 has been validated in the general population.
**Beck Depression Inventory-II**	21-item, self-rated inventory (questions scored 0–3). From validation in the general population: 0–9 minimal depression; 10–18 mild depression; 19–29 moderate depression; 30–63 severe depression.
**ANXIETY SCREENING INSTRUMENTS**	
**Beck Anxiety Inventory**	21-item, self-rated inventory (questions scored 0–3). From validation in the general population: 0–7 minimal anxiety; 8–15 mild anxiety; 16–25 moderate anxiety; 26–63 severe anxiety.
**Spielberger State-Trait Anxiety Inventory (STAI)**	40-item, self-rated inventory (questions scored 1–4) measuring state anxiety (S-Anxiety), or anxiety about an event, and trait anxiety (T-Anxiety), or anxiety level as a personal characteristic. Cut-off 39–40 has been suggested to detect clinically significant symptoms for the S-Anxiety scale.
**COMBINED SCREENING INSTRUMENTS**	
**Hospital Anxiety Depression Scale (HADS)**	14-item self-rated instrument (questions scored 0–3), 7 targeting anxiety (HADS-A) and 7 targeting depression (HADS-D). In the general population, a score of ≥8 on either of the two subscales suggests depression or anxiety.
**Toronto Western Spasmodic Torticollis**	Section of the Comprehensive Cervical
**Rating Scale-Psychiatric (TWSTRS-PSYCH)**	Dystonia Rating Scale that assesses depressive and anxiety symptoms, and which has been validated only in patients with cervical dystonia. It is a 6-item, self-report assessment instrument with questions scored from 0 to 4.
**Cervical Dystonia Impact Profile-58** (selected questions)	Selected screening questions on depression and anxiety. 1. How often do you feel anxious due to your neck problem? 2. How often do you feel down or depressed due to your neck problem? 3. How often do you feel frustrated due to your neck problem?
**Dystonia Non-Motor Symptoms Questionnaire** (selected questions)	Four screening questions on depression and anxiety (questions 5 to 8).

**Table 2:** Measures of diagnostic accuracy reported at the optimal cut-off point for each depression/anxiety self-administered screening tool used in the cervical dystonia cohort. BDI-II: Beck Depression Inventory-II; PHQ-9: Patient Health Questionnaire-9; HADS: Hospital Anxiety Depression Scale (D: depression; A: Anxiety); TWSTRS-PSYCH: Toronto Western Spasmodic Torticollis Rating Scale-Psychiatric scale; CDIP-58: Cervical Dystonia Impact Profile-58; DNMS-Quest: Dystonia Non-Motor Symptom Questionnaire; BAI: Beck Anxiety Scale; STAI: State-Trait Anxiety Inventory.

	Screening tool	Optimal cut-off	Prev, %	AUC, % Overall performance (adjusted)	95 % CI Overall performance (adjusted)	Se, %	Sp, %	PPV, %	NPV, %	LR+	LR-

**DEPRESSION**	**BDI-II**	19	18.6 %	91.2 %	84.8–98.8	87.5 %	87.1 %	35.9 %	98.2 %	6.79	0.14
	**PHQ-9**	7	28.6 %	85.4 %	77–94.8	87.5 %	76.3 %	23.3 %	98 %	3.69	0.16
	**HADS-D**	7	23.8 %	88.3 %	82.6–94	93.7 %	82 %	30 %	98.8 %	5.2	0.08
	**TWSTRS-PSYCH**	7	29.5 %	88.3 %	81.5–95.1	87.5 %	75.3 %	20.5 %	98.5 %	3.54	0.17
	**CDIP-58 selected questions**	6	30.5 %	83.3 %	73.8–92.8	81.2 %	73.7 %	21.9 %	97.9 %	3.09	0.25
	**DNMS-Quest (questions 5–8)**	3	24.3 %	84.9 %	74.9–95	81.2 %	80.4 %	27.5 %	98.1 %	4.15	0.23
**ANXIETY**	**BAI**	12	34.3 %	79.7 %	70–89.2	77.3 %	70.7 %	32.6 %	95.2 %	2.64	0.32
	**STAI**	89	66.6 %	53.5 %	42–64.9	77.3 %	34.6 %	12.9 %	96.4 %	1.18	0.66
	**HADS-A**	7	38.6 %	82.3 %	73–91.5	86.3 %	67 %	23.5 %	97.7 %	2.62	0.2
	**TWSTRS-PSYCH**	6	34.8 %	75.9 %	66.6–85.2	72.7 %	69.7 %	21.8 %	95.6 %	2.4	0.39
	**CDIP-58 selected questions**	5	36.2 %	76 %	66.1–85.7	72.7 %	68.1 %	21.1 %	95.5 %	2.28	0.40
	**DNMS-Quest (questions 5–8)**	2	41.4 %	71.9 %	61.2–82.5	72.7 %	62.2 %	18.4 %	95.1 %	1.93	0.44
**DEPRESSION and/or ANXIETY**	**TWSTRS-PSYCH**	6	16.2 %	81.8 %	74.7–89	75.8 %	72.9 %	35.6 %	94.2 %	2.79	0.33
	**CDIP-58 selected questions**	5	16.2 %	80.4 %	72.6–88.2	75.8 %	71.2 %	34.2 %	94 %	2.63	0.34
	**DNMS-Quest (questions 5–8)**	2	16.2 %	77.5 %	68.8–86.2	78.8 %	65.5 %	31.0 %	94.3 %	2.29	0.32

## Appendix A. Supplementary data

Supplementary data to this article can be found online at https://doi.org/10.1016/j.parkreldis.2025.107891.
