# Supplementary material for: Diagnostic accuracy of screening tools for depression and anxiety in cervical dystonia
Source: Parkinsonism Relat Disord. Author manuscript; Available in PMC 2025 Oct 30. (PMC12573120; doi:10.1016/j.parkreldis.2025.107891)
Supplement: Supplementary Table 1 [file NIHMS2118172-supplement-Supplementary_Table_1.docx]

**Supplementary Table 1.** Descriptive statistics of the study population of patients with idiopathic cervical dystonia.

| **Recruitment site or demographic or clinical variable** | **Descriptive statistics** |
| --- | --- |
| Recruitment centre  University of Calgary  Rocky Mountain Regional VA Medical Center, Aurora, CO  Washington University School of Medicine in St. Louis, St. Louis, MO  Virginia Commonwealth University, Richmond, VA  Emory University, Atlanta, GA  Westmead Hospital, Sydney  Baylor College of Medicine, Houston, TX  University of New Mexico, Albuquerque, NM  University of Florida, Gainesville, FA  Cardiff University | 49  20  19  21  20  20  20  8  13  20 |
| Age in years (mean ± standard deviation) | 64 ± 11.4 |
| Sex (Females/Males, absolute numbers) | 158/52 |
| Years of education  Median  <12 (absolute number, percentage)  13-16 (absolute number, percentage)  >16 (absolute number, percentage) | 16  41 (19.5%)  99 (47.1%)  70 (33.3%) |
| Anatomical spread of dystonia (data available for 190 of the 210 patients)  Focal cervical dystonia  Segmental/multifocal dystonia | 109 (57.4%)  81 (42.6%) |
| Stable ongoing treatments for depression and/or anxiety (absolute numbers, percentage)  Psychotherapy only  Pharmacotherapy only  Psychotherapy combined with pharmacotherapy  Benzodiazepines  Tricyclic antidepressants or serotonin selective reuptake inhibitors  Norepinephrine-serotonin selective reuptake inhibitors  Other psychotropic medications | 10 (4.8%)  49 (23.3%)  3 (1.4%)  17 (8.1%)  16 (7.6%)  8 (3.8%)  14 (6.7%) |
