# Supplementary figures and images for: Diagnostic accuracy of screening tools for depression and anxiety in cervical dystonia
Source: Parkinsonism Relat Disord. Author manuscript; Available in PMC 2025 Oct 30. (PMC12573120; doi:10.1016/j.parkreldis.2025.107891)

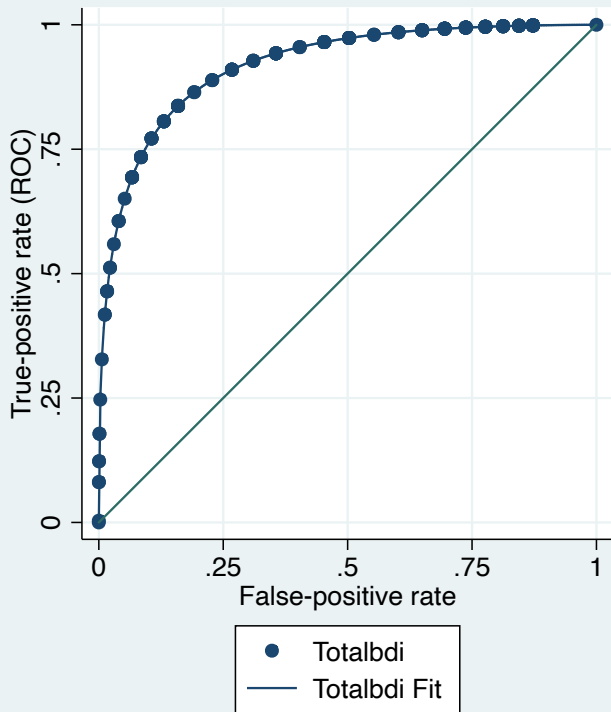

Supplement: Fig. S1 [file NIHMS2118172-supplement-Fig__S1.pdf]

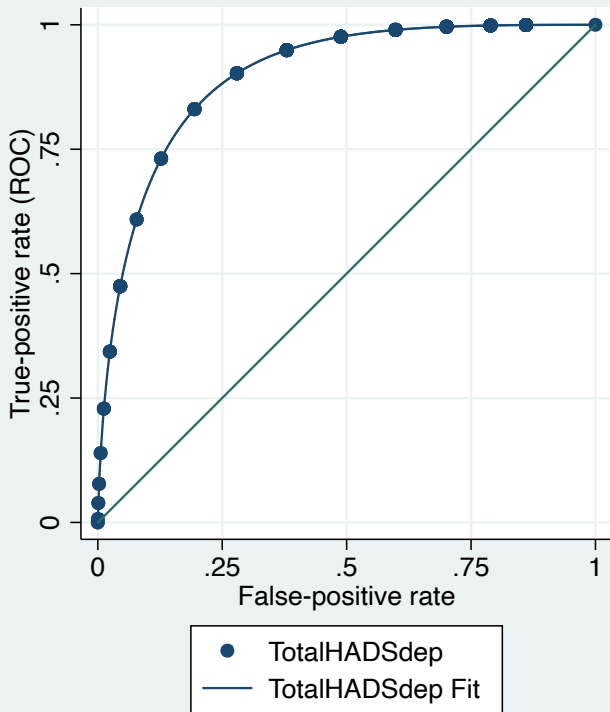

Supplement: Fig. S2 [file NIHMS2118172-supplement-Fig__S2.pdf]

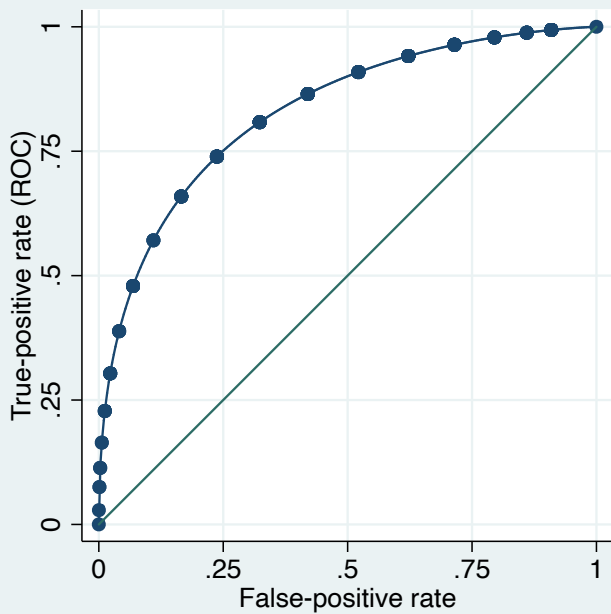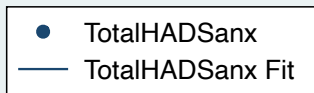

Supplement: Fig. S3 [file NIHMS2118172-supplement-Fig__S3.pdf]

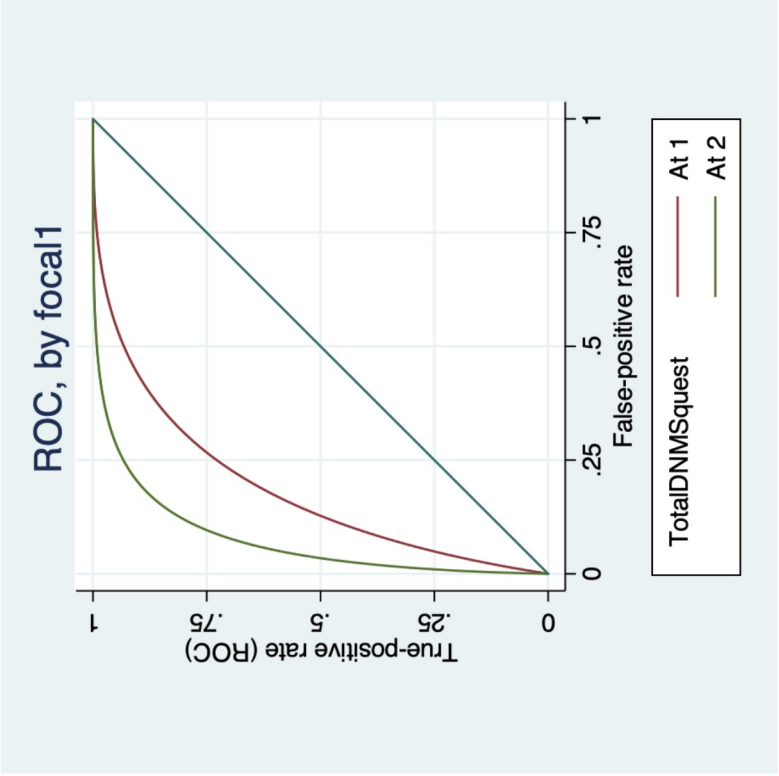

Coefficient = 0.89  
z = 1.99  
p = 0.046  
95% CI = 0.01-1.77

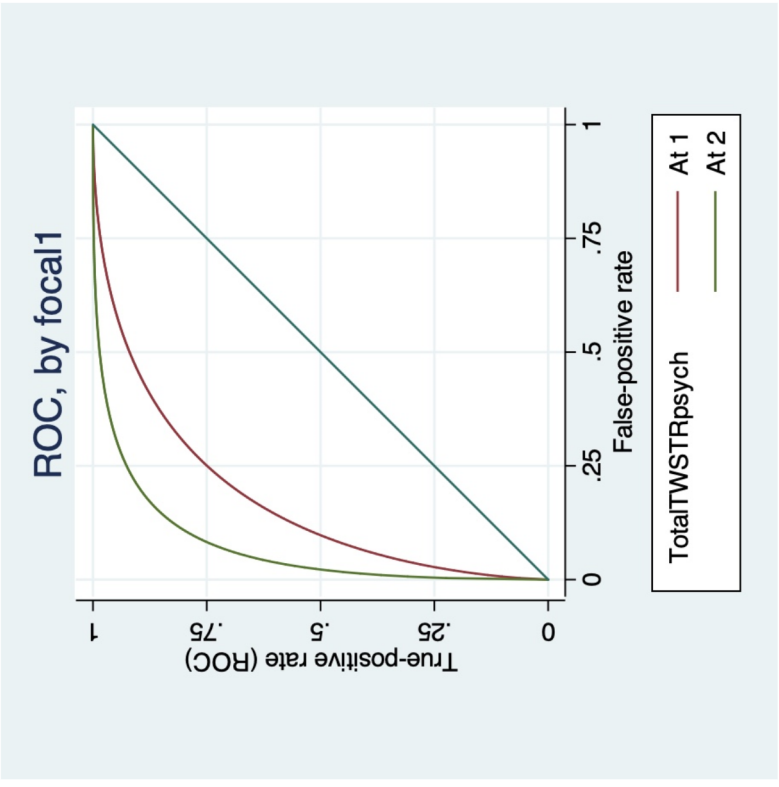

Coefficient = 0.77  
z = 1.51  
p = 0.13  
95% CI = -0.23-1.78

Supplement: Fig. S4 [file NIHMS2118172-supplement-Fig__S4.pdf]
